# Ferroptosis-Related Gene Signature Predicts Glioma Cell Death and Glioma Patient Progression

**DOI:** 10.3389/fcell.2020.00538

**Published:** 2020-07-09

**Authors:** Han-jie Liu, Hui-min Hu, Guan-zhang Li, Ying Zhang, Fan Wu, Xiu Liu, Kuan-yu Wang, Chuan-bao Zhang, Tao Jiang

**Affiliations:** ^1^Beijing Neurosurgical Institute and Beijing Tiantan Hospital of Capital Medical University, Beijing, China; ^2^Department of Neurosurgery, Beijing Tiantan Hospital, Capital Medical University, Beijing, China; ^3^Center of Brain Tumor, Beijing Institute for Brain Disorders, Beijing, China; ^4^China National Clinical Research Center for Neurological Diseases, Beijing, China; ^5^Chinese Glioma Genome Atlas Network (CGGA) and Asian Glioma Genome Atlas Network (AGGA), Beijing, China

**Keywords:** ferroptosis, prognosis, glioma, gene signature, dataset

## Abstract

Glioma is a fatal brain tumor characterized by rapid proliferation and treatment resistance. Ferroptosis is a newly discovered programmed cell death and plays a crucial role in the occurrence and progression of tumors. In this study, we identified ferroptosis specific markers to reveal the relationship between ferroptosis-related genes and glioma by analyzing whole transcriptome data from Chinese Glioma Genome Atlas, The Cancer Genome Atlas dataset, GSE16011 dataset, and the Repository of Molecular Brain Neoplasia Data dataset. Nineteen ferroptosis-related genes with clinical and pathological features of glioma were identified as highly correlated. Functional assays in glioma cell lines indicated the association of ferroptosis with temozolomide resistance, autophagy, and glioma cell migration. Therefore, the identified ferroptosis-related genes were significantly correlated with glioma progression.

## Introduction

Glioma is the most common primary malignant tumor in the central nervous system (Rasmussen et al., 2017). It can be graded from I to IV in accordance with the 2016 World Health Organization (WHO) classification (Louis et al., 2016). Owing to high proliferative rate, heterogeneity of tumor cells and diffuse infiltrating property, high-grade glioma is difficult to completely removed by surgery (Lara-Velazquez et al., 2017; Manrique-Guzman et al., 2017; Ferguson and McCutcheon, 2018). Moreover, chemotherapy resistance often occurs and leads to treatment failure and tumor recurrence (Osuka and Van Meir, 2017). Glioblastoma (WHO grade IV glioma, GBM) has a median overall survival (OS) of only 14.6 months (Lara-Velazquez et al., 2017). Molecular markers, such as O6-methylguanine-DNA methyltransferase (MGMT) methylation, codeletion of the short arm of chromosome 1 and the long arm of chromosome 19 (1p/19q), mutations in isocitrate dehydrogenase (IDH), mutations in alpha thalassemia/mental retardation syndrome X-linked, and epidermal growth factor receptor are being used in molecular pathological diagnosis, treatment options, and prognostic evaluation with glioma patients (Zeng et al., 2015). These molecular markers play a central role in regulating tumor cell proliferation and death (Ceccarelli et al., 2016; Stupp et al., 2019). Numerous glioma therapies targeting these molecular markers are used in clinical trials, but few have ultimately succeeded. Thus, novel targets for glioma therapy need to be identified.

Previous studies have confirmed that programmed cell death (PCD) is related to tumorigenesis (Labi and Erlacher, 2015), progression (Lee et al., 2018) and metastatic processes (Verschooten et al., 2012). Cancer treatment ultimately aims to induce cell-specific PCD in tumor tissues. Ferroptosis, characterized by iron-dependent lipid peroxide accumulation, is a newly discovered PCD distinct from traditional apoptosis or autophagic cell death or necrosis (Dixon et al., 2012). However, the roles of ferroptosis in tumor functions have yet to be elucidated. Notably, various studies have confirmed the pivotal role of ferroptosis in tumor development and therapies (Shen et al., 2018; Gan, 2019; Liang et al., 2019; Stockwell and Jiang, 2019). Ferroptotic regulatory genes such as P53 (Junttila and Evan, 2009), FANCD2 (Han et al., 2017), GPX4 (Liu H. et al., 2018), HSPB1 (Arrigo and Gibert, 2012), and DPP4 (Enz et al., 2019) are closely correlated to tumorigenesis and progression. Increasing evidence has shown that numerous tumor cells, including ovarian cancer cells (Carbone and Melino, 2019), adrenocortical carcinomas cells (Belavgeni et al., 2019), pancreatic cells (Eling et al., 2015), and hepatocellular carcinoma cells (Yang et al., 2014), are sensitive to ferroptosis. Moreover, combined with erastin, a ferroptosis inducer, chemotherapeutic drugs can improve their curative effect on GBM cells (Chen et al., 2015), acute myeloid leukemia cells (Yu et al., 2015), lung cancer cells (Guo et al., 2018), ovarian cancer cells (Sato et al., 2018), and gastric cancer cells (Zou et al., 2016). Therefore, ferroptosis can be a potential target for cancer therapy.

In the present study, we analyzed the differential expression of ferroptosis-related genes in glioma samples to identify the enriched pathways and their biological functions. We determined that ferroptosis-related genes were associated with the prognosis of glioma. Using the 19 identified ferroptosis-related genes, we accurately predicted the outcome for glioma patients. The underlying mechanisms were ultimately confirmed in *in vitro* studies. Overall, our data suggest that ferroptosis-related genes play pivotal roles in glioma progression and are potential prognostic markers and therapeutic targets for glioma.

## Materials and Methods

### Datasets

All datasets used in this study were available to the public. Expression RNA-seq data and clinical and molecular information of patients were obtained from the Chinese Glioma Genome Atlas (CGGA) dataset^1^ as a training set. The Cancer Genome Atlas (TCGA) dataset, the GSE16011 dataset, the Repository of Molecular Brain Neoplasia Data (REMBRANDT) dataset, and associated clinical information were obtained from the TCGA official website^2^, GEO website^3^, and GlioVis website^4^ as validation sets. The characteristics of the glioma patients in these 4 datasets—OS, age, gender, WHO grade, TCGA subtypes, and molecular pathological features—have been recorded in previous studies (Gravendeel et al., 2009; Bao et al., 2014; Tomczak et al., 2015; Gusev et al., 2018) (Table 1).

**TABLE 1:** Characteristics of patients in CGGA, TCGA, GSE16011, and REMBRANDT datasets.

**Characteristic**	***N***	**CGGA**	**TCGA**	**GSE16011**	**REMBRANDT**
Total cases	1783	325	699	284	475
**Gender**					
Male	974	203	368	182	221
Female	608	122	268	92	126
**Age (years)**					
≤40	356	31	246	79	
>40	795	208	390	197	
**Grade**					
I	8			8	
II	455	109	223	24	99
III	486	72	245	85	84
IV	605	144	168	159	134
**Subtype**					
Classical	256	74	92		90
Mesenchymal	313	68	105		140
Proneural	514	102	250		162
Neural	278	81	115		82
**IDH Status**					
Mutation	666	142	443	81	
Wildtype	554	168	246	140	

### Reagents

Dulbecco’s modified Eagle’s medium (DMEM), and fetal bovine serum (FBS) were purchased from Gibco (Thermo Fisher Scientific, Inc., Waltham, MA, United States). Erastin (Catalog No. S7242), a ferroptosis activator, was purchased from Selleck Chemicals LLC (Houston TX, USA). Antibodies specific for PD-L1 (ab213524) were supplied by Abcam (Cambridge, MA, United States). PARP (#9542) and LC3A/B (#12741) were purchased from Cell Signaling Technology (Danvers, MA, United States). β-actin (66009-1-Ig) was supplied by Proteintech (Rosemont, United States).

### Cell Culture

The human glioma cell line U87MG was obtained from the Cell Resource Center, Peking Union Medical College (Beijing, China), and U251MG was provided by the American Type Culture Collection (Manassas, VA, United States). Temozolomide (TMZ)-resistant U87MG cells (U87TR) and TMZ-resistant U251MG cells (U251TR) of GBM sub-cell lines, were established by repetitive exposure to increasing TMZ concentrations *in vitro* in our laboratory. Cells were cultured in the DMEM culture medium supplemented with 10% FBS with a standard humidified incubator under 5% CO2 at 37°C.

### RNA Sequencing of Glioma Cell Lines

Total RNA from U251MG, U87MG, U251TR, and U87TR cell lines were extracted and quantified. Up to 3 μg RNA per sample was used as the input material for library preparation. The library preparation was conducted using the standard Illumina HiSeq platform, and 150 bp paired-end reads from transcriptome sequencing were generated. After quality control procedures, the data were mapped to the human hg19 reference by STAR (Dobin et al., 2013). The expression level (Fragments per Kilobase Million, FPKM) of each gene was calculated based on the length of the gene and the read count mapped to this gene The RNA seq dataset was submitted to https://figshare.com/s/7af369b94634a51b11f1 for reference.

### Cell Viability Assay

Cell Counting Kit-8 Assay Kits (CCK-8; Dojindo, Kumamoto, Japan) were used to determine cell viability. The cells were seeded at a density of 2 × 103 cells/well in 96-well plates and were incubated with serum-containing media for 24 h. The cells were treated with erastin at varying concentrations (0, 10, 20, 30, 40, and 50 μM) on U251MG, U251TR, U87MG, and U87TR cells for 24 h. The medium was replaced with 100 μL of the fresh medium, and 10 μL of the CCK-8 solution was added to each well. Blank controls without cells were prepared. Subsequently, 100 μL of each sample was transferred to a new 96-well plate and was analyzed with the plate reader Infinite 200 PRO (Tecan Life Sciences). The absorbance values were determined at 450 nm.

### Western Blot Assay

The procedures for Western blot were described in detail in a previous study (Liu H. J. et al., 2018). In the current study, after incubation with primary antibodies, the HRP labeled anti-rabbit or anti-mouse secondary antibody was used for incubation at room temperature for another period of 1 h. Specific protein bands were detected using an ECL Western blotting kit, following the recommended procedure. β-actin was used as an internal control for sample loading and standardization.

### Migration Assay

The Transwell system (24 wells, 8 μm pore size with a polycarbonate membrane) was used for *in vitro* migration assays. The U251MG, U251TR, U87MG, and U87TR cells were pretreated with erastin (50 μM) or without erastin. A total of 1 × 10^5^ cells were suspended in 100 μL serum-free medium and then added to the upper chamber. The medium containing 10% FBS and erastin at different concentrations was added to the lower chamber. The cells in the upper chamber were carefully wiped using a cotton swab after 2 h for the U87MG and U87TR cells or 7 h for the U251MG and U251TR cells. The cells that were attached to the lower surface of the filter were fixed with 4% paraformaldehyde and then stained with crystal violet. The migrated cells on the lower surface of the membrane filter were photographed using an Axio Observer3 microscope (Carl Zeiss), and 5 randomized fields were counted using the Image J software. All experiments were repeated three times.

### Gene Signature Building

We chose 40 genes, which were verified to be involved in ferroptosis (Stockwell et al., 2017). Univariate Cox analysis was first performed, and genes with P values less than or equal to 0.0001 were retained. We then developed a risk score model for each patient on the basis of the expression level of the 19 genes, and their regression coefficients were derived from the univariate Cox regression analysis. The risk score was expressed as (expr_gene__1_ × coefficient_*gene*__1_) + (expr_*gene*__2_ × coefficient_*gene*__2_) + ⋯ + (expr_*gene*__19_ × coefficient_*gene*__19_). The regression coefficients derived from the training set were then applied into the three other validation sets (TCGA, GSE16011, and REMBRANDT datasets) to calculate the risk scores.

### Statistical Analysis

The receiver operating characteristic (ROC) curve and a nomogram were used to show the predictive accuracy of the gene signature with the R package “survival ROC” (Heagerty et al., 2000) and “rms” (Feng et al., 2017). The area under the curve (AUC) of the ROC curves, concordance index (C-index), and calibration curves of nomograms were calculated. Gene ontology (GO) analysis and Kyoto Encyclopedia of Genes and Genomes (KEGG) pathway analysis were applied in DAVID^5^ (Dennis et al., 2003) for functional annotation of the genes positively correlated with risk score in the 4 cohorts. Gene Set Variation Analysis (GSVA) was conducted to detect the difference in expression with risk score by using the R package GSVA (Hanzelmann et al., 2013). We classified the patients in each dataset into two groups (high-risk-score and low-risk-score groups) in accordance with the median risk score of the ferroptosis-related gene signature. Kaplan–Meier curves and the log-rank test were used to assess the differences in OS between two groups. Independent prognostic factors were identified by univariate and multivariate Cox regression analysis. All statistical analyses were conducted using SPSS, GraphPad Prism 7, or the R software. *P* < 0.05 was considered statistically significant.

## Results

### Identification of 19 Ferroptosis-Related Genes

**FIGURE 1:**
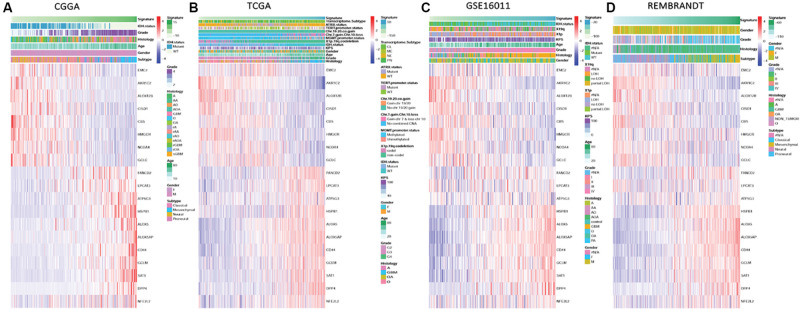
Ferroptosis-related genes expression profiles and correlation between signature risk score, gene expression and clinical or molecular pathological features in CGGA, TCGA, GSE16011, and REMBRANDT datasets. **(A–D)** Heatmaps show the different expression levels of 19 ferroptosis-related genes horizontally clustered in the CGGA dataset and clinical or molecular pathological features ranked by risk score of the signature of 19 ferroptosis-related genes in CGGA **(A)**, TCGA **(B)**, GSE16011 **(C)**, and REMBRANDT **(D)** datasets. Chinese Glioma Genome Atlas (CGGA); The Cancer Genome Atlas (TCGA); The Repository of Molecular Brain Neoplasia Data (REMBRANDT); isocitrate dehydrogenase (IDH); methylguanine methyltransferase (MGMT); Karnofsky Performance Status (KPS); classical (CL), mesenchymal (MES), neural (NE), and proneural (PN); alpha thalassemia/mental retardation syndrome X-linked (ATRX); telomerase reverse transcriptase (TERT); chromosome (Chr); wild type (WT); codeletion (codel); female (F); male (M); grade (G); astrocytoma (A); oligodendroglioma (O); oligoastrocytoma (OA); anaplastic astrocytoma (AA); anaplastic oligodendroglioma (AO); anaplastic oligodendroglioma (AOA); pilocytic astrocytoma, (PA); glioblastoma multiforme (GBM); loss of heterozygosity (LOH).

To characterize the ferroptosis-related gene expression pattern in gliomas, we examined the RNA-seq data of glioma patients from 4 datasets (CGGA, TCGA, REMBRANDT, and GSE16011). First, univariate Cox regression analysis was performed to identify the genes related to patient survival in the CGGA dataset. Subsequently, 19 of the 40 ferroptosis-related genes were selected in glioma patients (SAT1, ATP5G3, FANCD2, HSPB1, HMGCR, CBS, GCLC, GCLM, CD44, ALOX12B, ALOX5AP, CISD1, NFE2L2, EMC2, ALOX5, DPP4, AKR1C2, LPCAT3, and NCOA4, *P* < 0.0001). A gene-based prognostic model was then established to evaluate the risk of each patient as described in the methods. Details on the 19 genes are presented in Table 2. The risk scores of each patient in these four datasets (CGGA, TCGA, REMBRANDT, and GSE16011) were calculated. Heat maps were shown to present the different expression levels of the 19 selected genes and clinical information ordered by risk score in the CGGA (Figure 1A), TCGA (Figure 1B), GSE16011 (Figure 1C), and REMBRANDT (Figure 1D) datasets. In the CGGA cohort, with an increase in risk score, the expression levels of EMC2, AKR1C2, ALOX12B, CISD1, CBS, HMGCR, NCOA4, and GCLC were distinctly downregulated. Meanwhile, the expression levels of FANCD2, LPCAT3, ATP5G3, HSPB1, ALOX5, ALOX5AP, CD44, GCLM, SAT1, DPP4, and NFE2L2 were upregulated. Clinical and molecular features, such as WHO grade, age, classical subtypes, mesenchymal subtypes, and IDH wild types were enriched in high-risk-score gliomas. The other three cohorts showed similar patterns of genes and clinical information. Notably, the MGMT gene with promoter unmethylation was enriched in high-risk-score glioma in the TCGA dataset. These results suggest that the risk score of signatures of the 19 ferroptosis-related genes positively correlated with glioma malignancy, and the expression levels of the 19 genes in the gliomas exhibit a pattern similar to those of the other three datasets.

**TABLE 2:** *P*-value and regression coefficient of 19 ferroptosis-related genes.

**Gene**	***p*-value**	**Coefficient**
SAT1	9.19E-19	0.03262
ATP5G3	2.47E-19	0.690305
HSPB1	6.11E-17	0.545296
FANCD2	9.25E-17	1.059988
HMGCR	2.03E-12	–1.84672
CBS	3.60E-11	–1.44061
GCLC	2.60E-10	–1.09568
GCLM	3.67E-10	0.082152
CD44	5.78E-10	2.363275
ALOX12B	1.63E-08	–4.62635
ALOX5AP	1.01E-07	3.924738
CISD1	2.69E-07	–0.10964
NFE2L2	4.98E-07	0.815403
EMC2	7.77E-07	–0.04527
ALOX5	1.21E-06	6.23031
DPP4	1.00E-05	0.052881
AKR1C2	1.04E-05	–17.1096
LPCAT3	3.07E-05	0.04055
NCOA4	3.09E-05	–1.87573

### Association of Risk Scores With Patient Prognosis and Glioma Grade

**FIGURE 2:**
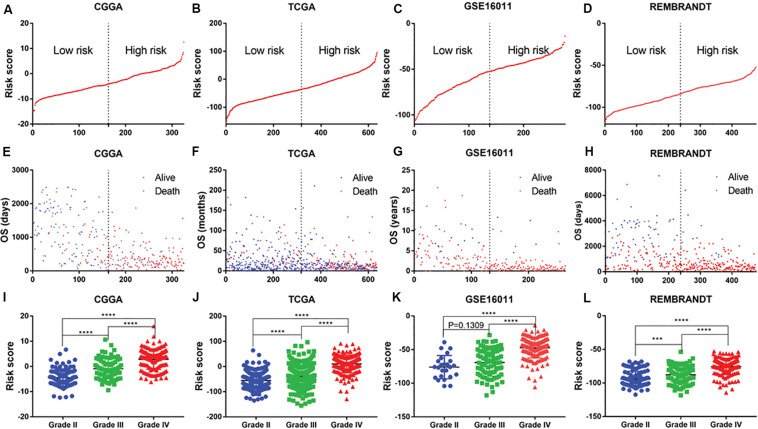
Distribution of risk score, OS, WHO grade in CGGA, TCGA, GSE16011 and REMBRANDT datasets. **(A–D)** mRNA risk score distribution and **(E–H)** overall survival (OS) status in the four datasets. **(I–L)** association of risk score with WHO grades. ***P < 0.001, ****P < 0.0001.

The distribution of risk scores in the CGGA, TCGA, GSE16011, and REMBRANDT datasets is presented (Figures 2A–D). Glioma patients were divided into low-risk and high-risk-score groups on the basis of their median risk scores. The OS of each patient was shown in the CGGA, TCGA, GSE16011, and REMBRANDT (Figures 2E–H) datasets. The patients with a low-risk-score had a markedly lower mortality rate than those with a high-risk-score in these four datasets. Meanwhile, with an increase in glioma grade, the risk score increased. The highest increase in risk score was found in the WHO grade IV patients, whereas the lowest increase in risk score was observed in the WHO grade II patients. The WHO grade III patients were assigned moderate risk scores in the CGGA, TCGA, GSE16011, and REMBRANDT datasets (Figures 2I–L). All results were significantly different, except for those in the GSE16011 dataset, which could be attributable to the limited number of WHO grade II patients.

### Validation of 19 Ferroptosis Related Gene Signature in Survival Using Kaplan–Meier Curves

**FIGURE 3:**
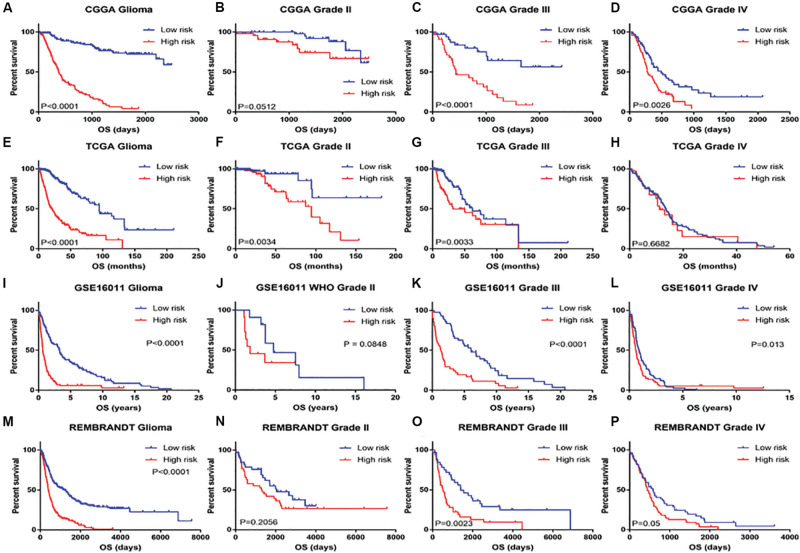
Kaplan–Meier survival analysis for glioma patients with low and high risk scores in CGGA, TCGA, GSE16011, and REMBRANDT datasets. Kaplan–Meier survival curve for glioma patients with a high risk score (red line) and a low risk score (blue line), classified as WHO grade II to IV in CGGA, TCGA, GSE16011, and REMBRANDT datasets.

Patients with different kinds of glioma, such as pan-glioma and WHO grade II–IV glioma, were divided into two groups based on their median risk scores. The Kaplan–Meier curve for the CGGA dataset showed that the high-risk patients had significantly shorter OS than low-risk patients in the glioma (Figure 3A), WHO grade II glioma (Figure 3B), WHO grade III (Figure 3C), and WHO grade IV glioma (Figure 3D) groups. Consistency of results was validated for the TCGA (Figures 3E–H), GSE16011 (Figures 3I–L), and REMBRANDT (Figures 3M–P) datasets. Moreover, univariate Cox regression and multivariate Cox regression of the signature of the 19 ferroptosis-related genes were performed in the CGGA dataset (*p* < 0.001, univariate Cox regression; *p* < 0.05, multivariate Cox regression, Table 3). The independence of the clinical prognostic significance of the signature in glioma was verified. The risk score showed significance in both univariate Cox regression and multivariate Cox regression. These consistent results were also validated in the TCGA (Table 4), GSE16011 (Table 5), and REMBRANDT (Table 6) datasets.

**TABLE 3:** Univariate and multivariate Cox analysis of signature in CGGA dataset.

**Variable**	**Univariate analysis**		**Multivariate analysis**	
	**HR (95% CI)**	***p*-value**	**HR (95% CI)**	***p*-value**
Signature	1.212 (1.174–1.251)	< 0.001	1.182 (1.126–1.242)	< 0.001
Age	1.038 (1.023–1.054)	< 0.001	0.987 (0.969–1.005)	0.155
WHO Grade	3.477 (2.716–4.452)	< 0.001	2.388 (1.739–3.280)	< 0.001
Chemotherapy	1.378 (0.963–1.971)	0.079	0.648 (0.439–0.958)	0.030
Radiotherapy	0.429 (0.296–0.622)	< 0.001	0.383 (0.258–0.570)	< 0.001
				

**TABLE 4:** Univariate and multivariate Cox analysis of signature in TCGA dataset.

**Variable**	**Univariate analysis**		**Multivariate analysis**	
	**HR (95% CI)**	***p*-value**	**HR (95% CI)**	***p*-value**
Signature	1.017 (1.014–1.020)	< 0.001	1.006 (1.001–1.010)	0.009
WHO Grade	4.794 (3,798–6,051)	< 0.001	2.332 (1.645–3.305)	< 0.001
Age	1.073 (1.061-1.084)	< 0.001	1.037 (1.021–1.054)	< 0.001
KPS	0.954 (0.944–0.965)	< 0.001	0.986 (0.973–1.000)	0.045

**TABLE 5:** Univariate and multivariate Cox analysis of signature in GSE16011 dataset.

**Variable**	**Univariate analysis**		**Multivariate analysis**	
	**HR (95% CI)**	***p*-value**	**HR (95% CI)**	***p*-value**
Signature	1.028 (1.021–1.035)	< 0.001	1.025 (1.016–1.035)	< 0.001
Age	1.042 (1.032–1.053)	< 0.001	1.028 (1.014–1.042)	< 0.001
WHO Grade	2.574 (2.047–3.240)	< 0.001	1.731 (1.256–2.386)	0.001
Chemotherapy	1.571 (1.013–2.436)	0.044	1.420 (0.895-2.254)	0.136
Radiotherapy	0.983 (0.725–1.333)	0.912	0.947 (0.336–2.671)	0.918
KPS	0.980 (0.973–0.986)	< 0.001	0.987 (0.978–0.997)	0.009

**TABLE 6:** Univariate and multivariate Cox analysis of signature in REMBRANDT dataset.

**Variable**	**Univariate analysis**		**Multivariate analysis**	
	**HR (95% CI)**	***P*-value**	**HR (95% CI)**	***P*-value**
Signature	1.047 (1.037–1.056)	< 0.001	1.034 (1.020–1.047)	< 0.001
WHO Grade	1.694 (1.422–2.019)	< 0.001	1.272 (1.037–1.559)	0.021

### Prognostic Validity of the Signature of 19 Ferroptosis-Related Genes for Glioma

**FIGURE 4:**
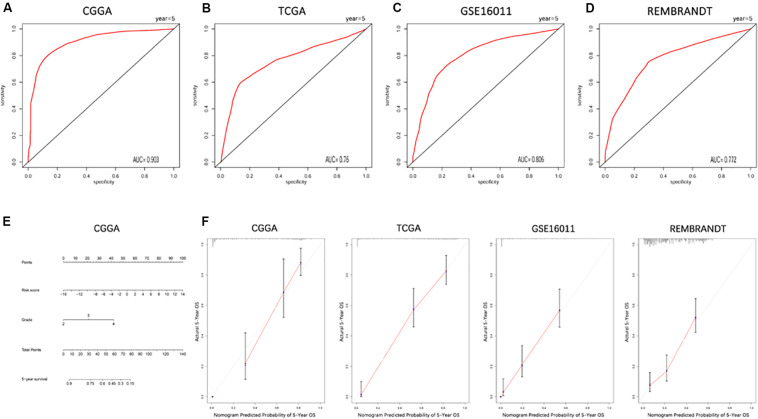
ROC curves and a nomogram of the signature of 19 ferroptosis-related genes predicting survival in glioma patients. **(A–D)** ROC curves showing the sensitivity and specificity of predicting 5-year survival with the ferroptosis-related gene signature in CGGA **(A)**, TCGA **(B)**, GSE16011 **(C)**, and REMBRANDT **(D)** datasets. **(E)** A nomogram for predicting 5-year cancer-specific survival in the CGGA dataset. **(F)** Calibration curves for the nomogram predicting 5-year survival in glioma patients in the CGGA, TCGA, GSE16011, and REMBRANDT datasets.

The ROC curve was plotted to illustrate the sensitivity and specificity of risk score in predicting the 5-year survival of glioma patients. The signature of the 19 ferroptosis-related genes exhibited striking prognostic validity, with AUC values of 0.903, 0.76, 0.806, 0.772 in CGGA, TCGA, GSE16011, and REMBRANDT (Figures 4A–D). A 5-year survival nomogram prediction model was then built with independent prognostic parameters for the OS of patients in the CGGA dataset (Figure 4E). The C-indices were 0.79 in the primary CGGA dataset, 0.849 in the TCGA dataset, 0.75 in the GSE16011 dataset, and 0.653 in the REMBRANDT dataset as validation. Meanwhile, the calibration plot for the probability of 5-year survival showed an optimal agreement between observation and prediction in these datasets (Figure 4F). These results indicated that the signature of the 19 ferroptosis-related genes was a reliable prognostic indicator in glioma patients.

### Functional Annotation of the Signature of 19 Ferroptosis-Related Genes

**FIGURE 5:**
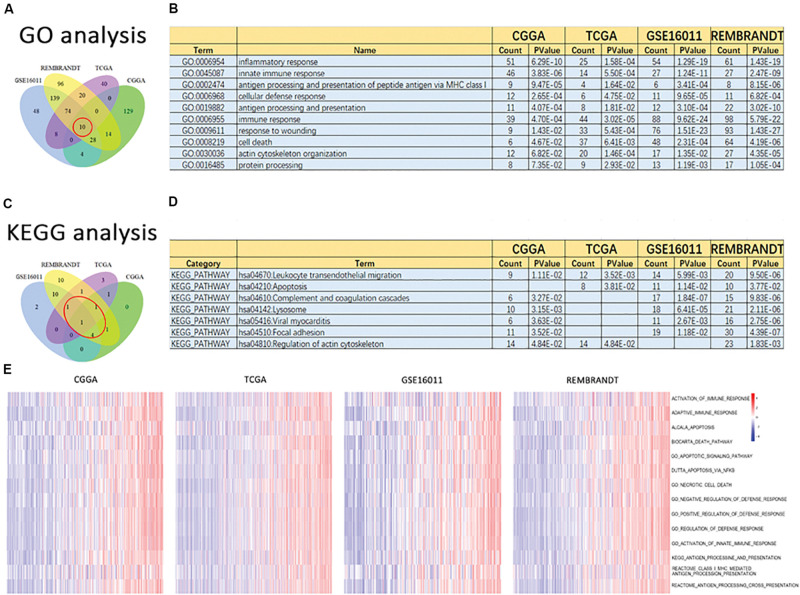
Altered functional characteristics related to the signature of 19 ferroptosis-related genes. Functional annotation of genes positively correlated with the risk score using the GO terms of biological processes, the KEGG pathway, and GSVA in the CGGA, TCGA, GSE16011, and REMBRANDT datasets. **(A)** Wayne Diagram shows the results of GO analysis in the four datasets. **(B)** Intersection of GO terms in the four datasets. **(C)** Wayne Diagram shows the results of KEGG analysis in the four datasets. **(D)** Intersection of the KEGG pathways in the four datasets. **(E)** Heatmaps of the GSVA results in different gene sets in the four datasets.

To clarify the potentially functional characteristics of the signature of 19 ferroptosis-related genes in glioma, GO analysis and KEGG analysis were conducted. We first created a list of genes that were positively correlated with risk score, as revealed by Pearson correlation analysis in the CGGA (Pearson *R* > 0.6, *p* < 0.05), TCGA (Pearson *R* > 0.5, *p* < 0.05), GSE16011 (Pearson *R* > 0.6, *p* < 0.05), and REMBRANDT (Pearson *R* > 0.6, *p* < 0.05) datasets (Supplementary Table S1). We then explored the biofunctions of the genes in each dataset by GO analysis and KEGG analysis using DAVID Bioinformatics Resources 6.8. Genes with the p value of gene functions > 0.05 were excluded. The GO terms of the remaining genes in the CGGA (Supplementary Table S2), TCGA (Supplementary Table S3), GSE16011 (Supplementary Table S4), and REMBRANDT (Supplementary Table S5) datasets were listed. These gene functions were excluded from the intersection of these four datasets, as reflected in the Venn diagram of the resulting GO terms in these datasets (Figure 5A). Ten of the GO terms were included in all datasets (Figure 5B): inflammatory response, innate immune response, antigen processing and presentation of peptide antigen via MHC class I, cellular defense response, antigen processing and presentation, immune response, response to wounding, cell death, and actin cytoskeleton organization and protein processing. These functions play a key role in tumorigenesis and progression. KEGG analysis was performed based on the aforementioned gene list. Gene pathways with a *p*-value > 0.05 were excluded. The remaining gene pathways in the CGGA (Supplementary Table S6), TCGA (Supplementary Table S7), GSE16011 (Supplementary Table S8), and REMBRANDT (Supplementary Table S9) datasets are listed. The results of the KEGG pathway analysis in these datasets are reflected in the Venn diagram in Figure 5C. Seven cancer-related terms in KEGG were included in at least three datasets (Figure 5D), which was consistent with the GO results. The terms were hsa04670: leukocyte transendothelial migration, hsa04210: apoptosis, hsa04610: complement and coagulation cascades, hsa04142: lysosome, hsa05416: viral myocarditis, hsa04510: focal adhesion, and hsa04810: regulation of actin cytoskeleton. Subsequently, 14 gene sets related to the GO terms were applied to GSVA analyses for validation. Consistent with the GO results, heat maps showed that the risk score was positively associated with these gene sets in the CGGA, TCGA, GSE16011, and REMBRANDT datasets (Figure 5E). All aforementioned results suggest that the signature of the 19 ferroptosis-related genes is related to cancer biology, particularly cell death, migration, and immune-related functions.

### Association of Ferroptosis With Glioma Drug Resistance

**FIGURE 6:**
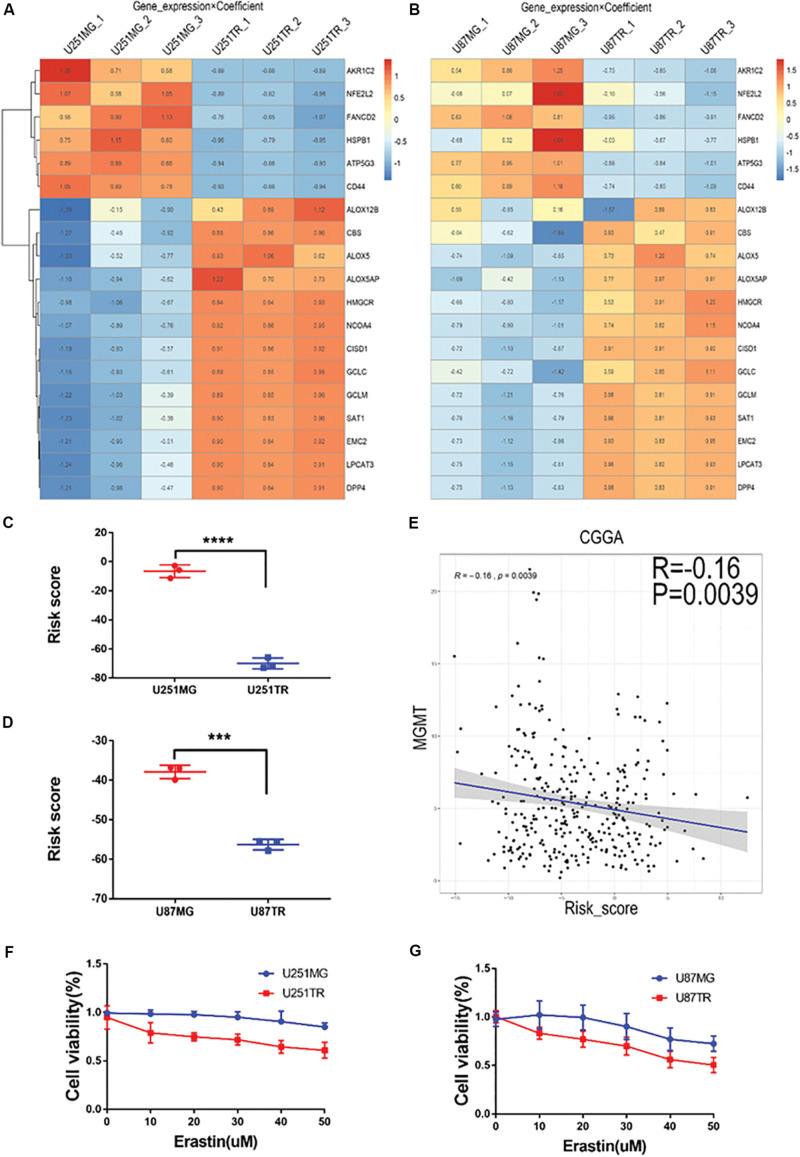
Relationship of risk score and glioma drug resistance and cell viability analysis after erastin treatment in U251 and U87 cells. **(A,B)** Heatmaps showing gene expression × coefficient as revealed by RNAseq in the glioma cell lines U251 **(A)** and U87 **(B)**. **(C,D)** Risk scores were significantly lower in the drug-resistant groups (U251TR and U87TR) than in the drug-sensitive groups (U251MG and U87MG). **(E)** Curve of the risk score and MGMT expression levels. Notably, low risk scores were correlated with high expression of MGMT. **(F,G)** Treatment with erastin at different concentrations (0, 10, 20, 30, 40, and 50 μM, 24 h) further inhibited cell viability, indicating the proliferation of glioma cells in TMZ-resistant lines (U251TR and U87TR) rather than in regular glioma cell lines (U251MG and U87MG).

To determine the relationship between ferroptosis and glioma drug resistance, we periodically treated glioma cell lines U87 and U251 with TMZ and constructed their corresponding drug-resistant strains U87TR and U251TR, respectively. The mRNA expression profiling technique was used to detect the expression levels of ferroptosis-related genes in different glioma cell lines. Changes in the expression pattern of the 19 ferroptosis-related genes were consistent (Figures 6A,B). Differences in the risk score of the characteristics of the ferroptosis-related genes were consistent as well. Meanwhile, the risk scores of the drug-resistant cells were lower than those of the normal tumor cells (Figures 6C,D). In the CGGA glioma dataset, the risk score of the signature of the 19 ferroptosis-related genes was negatively correlated to the expression level of the well-recognized gene MGMT for TMZ resistance (Figure 6E), verifying the results for the risk scores of resistant cells cultured *in vitro*. We used erastin to treat the normal and drug-resistant glioma cell lines at increasing doses and CCK-8 assay to evaluate cell proliferation activity. We found that the U87TR and U251TR cell lines were more sensitive to erastin, compared with the U87MG and U251MG cell lines (Figures 6F,G). These results indicate that compared with normal glioma cells, TMZ-resistant cells are more likely to induce ferroptosis. The lower their risk scores, the more likely the occurrence of ferroptosis.

### Relationship Between Ferroptosis and Autophagy and Apoptosis

**FIGURE 7:**
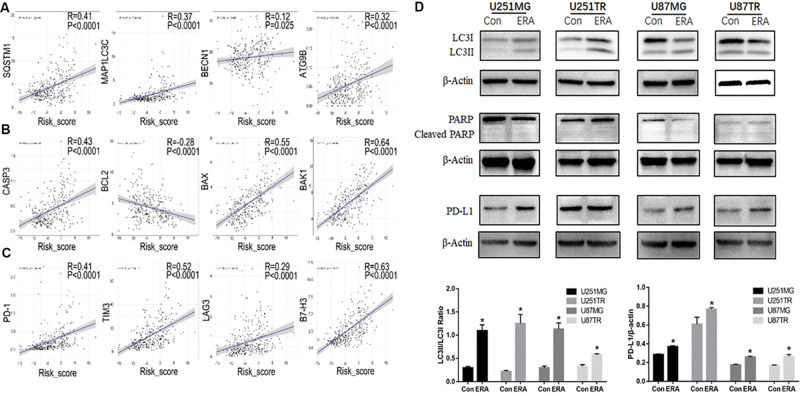
Relationship between risk scores of the ferroptotic signature and autophagy. **(A–C)** In the CGGA database, correlation analysis of ferroptosis-related gene risk scores and autophagy markers (**A**, SQSTM1, MAPLC3C, BECN1, and ATG9B), apoptosis markers (**B**, CASP3, BCL2, BAX, and BAK1), and immune checkpoint markers (**C**, PD-1, TIM3, LAG3, and B7-H3). **(D)** Western blot analysis showing the expression levels of LC3 PARP and its cleaved-PARP, and PD-L1 in U251 and U87 glioblastoma cell lines treated with erastin (50 μM for 24 h). Data are presented as mean ± SEM. *P < 0.05 compared with respective controls.

In the CGGA dataset, we analyzed the risk scores of the signature of the 19 ferroptosis-related genes and autophagy markers (SQSTM1, MAPLC3C, BECN1, and ATG9B), apoptosis markers (CASP3, BCL2, BAX, and BAK1), and immune checkpoint markers (PD-1, TIM3, LAG3, and B7-H3). We found that the highest risk scores were positively correlated with these markers, except BCL2 (Figures 7A–C). Moreover, we determined the relationship between erastin-induced ferroptosis and autophagy, apoptosis, and immune function in the GBM cell lines. Erastin at different concentrations (0–50 μM) was used to treat the U251 and U87 GBM cell lines. Erastin treatment increased the expression of LC3 (an autophagy marker) and PD-L1 (an immune checkpoint marker) (Figure 7D). However, treatment with erastin failed to change the expression level of cleaved PARP, an apoptotic molecule. These data suggest that erastin-induced ferroptosis is closely related to autophagy and immune function in tumor cells but not to apoptosis.

### Ferroptosis Is Positively Associated With Glioma Cell Migration

**FIGURE 8:**
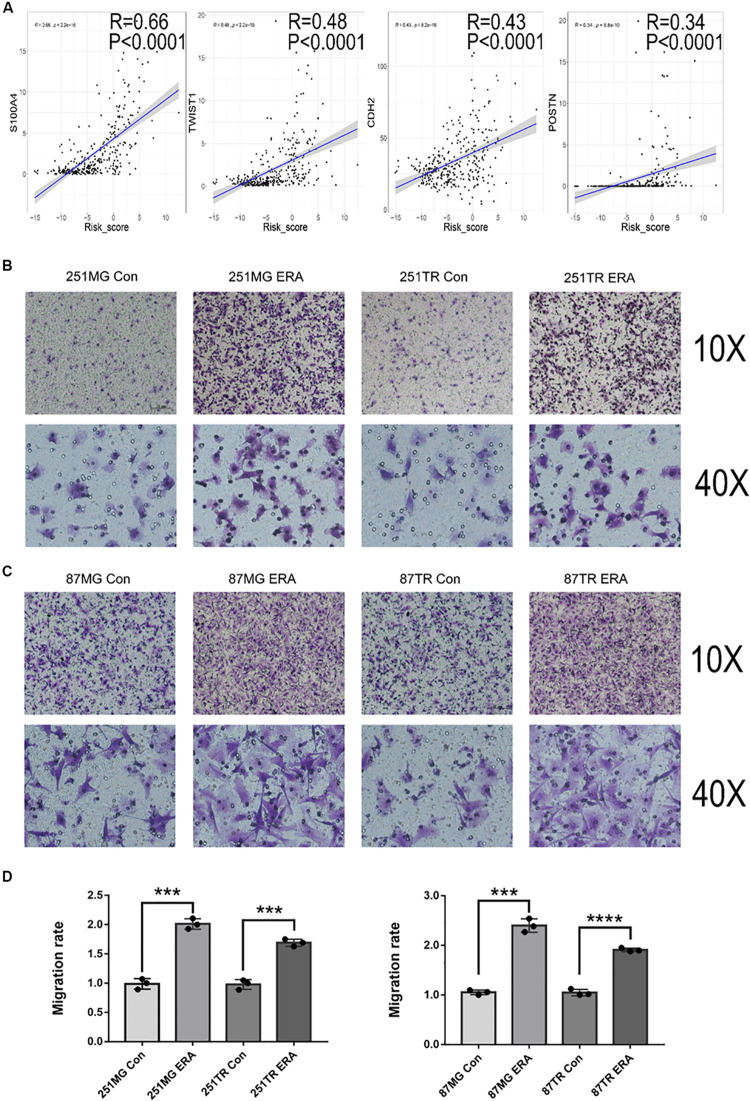
Ferroptosis is positively correlated with glioblastoma cell migration. **(A)** Analysis in the CGGA database showing the positive correlation between risk score and migration-related genes. **(B–D)** Images under a light microscope and a summary of data showing that treatment with erastin (50 μM) increased the migration of U251MG, U251TR, U87MG, and U87TR cells relative to those of vehicle-treated cells. Data are presented as mean ± SEM of three samples (***P < 0.001, ****P < 0.0001; ERA, erastin).

To determine the relationship between ferroptosis and glioma cell migration, we analyzed the relationship between the risk scores of the signature of 19 ferroptosis-related genes and migration-related genes. The risk score was positively correlated to the oncogenes S100A4, TWIST1, CDH2, and POSTN, which were critically involved in glioma migration and invasion (Figure 8A). We then determined the migration ability of glioma cell lines (U251 and U87) treated with a vehicle or erastin by using Transwell migration assay. Compared with the vehicle-treated U251 and U87 cells, the erastin-treated U251 and U87 cells increased in migratory ability (Figures 8B–D). These data suggest that ferroptosis enhances cell migration in glioma cells.

## Discussion

Selective induction of cancer cell death is the most effective anticancer therapy. Increasing evidence has shown that ferroptosis, a recently discovered PCD, plays a crucial role in tumorigenesis and cancer therapeutics. However, profiling of ferroptosis in glioma has yet to be clarified. In this study, we used high-throughput expression analysis to investigate variations in expression profiling of ferroptosis-related genes in glioma. On the basis of these analyses, we identified the signature of the 19 ferroptosis-related genes. We further explored tumor clinicopathological features and prognosis, which were closely related to tumorigenesis.

**FIGURE 9:**
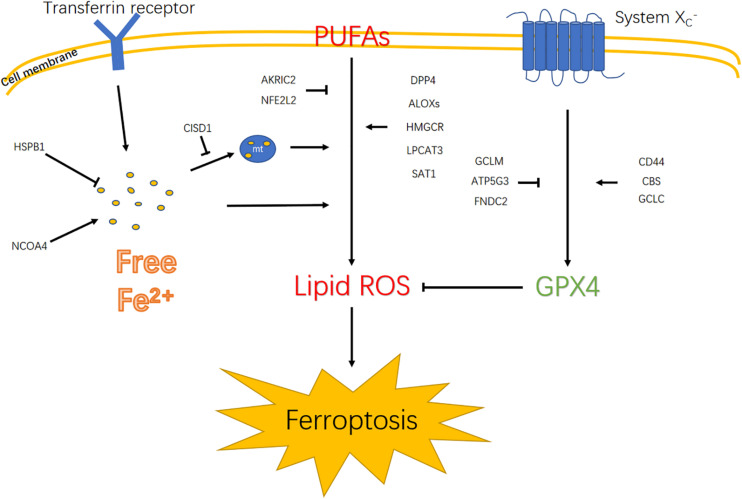
Diagram illustrates signaling pathways that trigger ferroptosis. Polyunsaturated fatty acids (PUFAs); mitochondrion (mt).

Previous studies suggest that ferroptotic cell death results from fatal lipid peroxidation (Gao et al., 2015). In this regard, the accumulation of intracellular iron caused by the depletion of ferritin or iron transporters and subsequent peroxidation are fundamental mechanisms that lead to the accumulation of lipid peroxides and ferroptosis (Stockwell and Jiang, 2019). Among the 19 identified genes related to ferroptosis, CISD1 alleviated iron accumulation in the mitochondria, and NCOA4 promoted intracellular iron accumulation, whereas HSPB1 decreased intracellular iron accumulation. Polyunsaturated fatty acyl tail-containing phospholipids, the substrate of lipid peroxidation, is considered essential for ferroptosis. Lipid peroxidation is negatively regulated by AKRIC2 and NFE2L2 but positively regulated by DPP4, ALOXs, HMGCR, LPCAT3, and SAT1. Malfunctioning of scavenging lipid peroxide also leads to ferroptosis. After being transported intracellularly by the system XC-, cystine may become cysteine, which is used to synthesize glutathione. GPX4 and GSH scavenge lipid peroxide to inhibit ferroptosis (Lewerenz et al., 2006). In the signature genes, CD44, CBS, and GCLC were positive regulators, whereas GCLM, ATP5G3, and FNDC2 were negative regulators for synthesizing GPX4. Thus, the coexistence of the aforementioned factors may trigger ferroptosis (Figure 9). In the current study, we found that higher risk scores were associated with worse prognosis in glioma patients. Moreover, high risk scores were insensitive to ferroptosis in glioma cell lines. To verify this finding, cell death pathways were shown to be frequently disturbed in human cancers (Bebber et al., 2020).

By focusing on the specific function of the 19 ferroptotic genes, previous studies have demonstrated that most of these genes played a pivotal role in cancer cells, including glioma. For instance, high expression of HSPB1 is correlated with a low survival rate in glioma patients. HSPB1 also enhances proliferation via SIRT2-mediated G6PD activation in glioma cells (Ye et al., 2016). The increased expression of SAT1 in GBM was related to the resistance of tumor cells to radiotherapy (Brett-Morris et al., 2014). During lung carcinogenesis, NFE2L2 accelerates progression via the KRAS signaling pathway (Satoh et al., 2013). Decreased expression of CBS promotes the formation of glioma tumors by increasing the HIF-2α protein levels and HIF-2 target gene expression (Takano et al., 2014). These genes are either positively or negatively related to the regulation of ferroptosis and are distinctly expressed in the high-risk-score or low-risk-score groups. This occurrence is consistent with their gene functions in cancers. Notably, not all 19 genes had expression levels consistent with their functions in previous reports. Busek et al. (2012) indicated that DPP4, which is highly expressed in gliomas with a high risk score, inhibits glioma cell growth independent of its enzymatic activity. Another study showed that DPP4 was related to the stemness of glioma stem cells (Sakamoto et al., 2019). Sohn et al. (2013) demonstrated that CISD1 that was highly expressed in human epithelial breast cancer cells suppressing CISD1 expression significantly inhibited cell proliferation and tumor growth. ARK1C2 was found to positively regulate proliferation in cancer cells (Ji et al., 2004). We found that ARK1C2 expression was low in high-risk-score groups. Therefore, the specific role of these genes in glioma has to be clarified.

We further demonstrated that the risk scores of the signature of the 19 ferroptosis-related genes were highly associated with the WHO tumor grades. The ROC curve generated using the risk scores of the signature of the 19 ferroptosis-related genes predicted patient OS. In addition, the signature of the 19 ferroptosis-related genes was independent of other clinical factors, including age, radiotherapy, grade, and chemotherapy. These results suggested that the activated process of ferroptosis in glioma cells were associated with improved survival of glioma patients.

Functional annotation of the signature of the 19 ferroptosis-related genes showed that biological functions such as immune response, cellular defense response, actin cytoskeleton organization, cell death, and protein processing could contribute to the poor clinical outcome of patients. KEGG analysis indicated that the signature of the 19 ferroptosis-related genes corresponding to biological functions were closely related to apoptosis, focal adhesion, regulation of actin cytoskeleton, leukocyte transendothelial migration, and lysosome pathways, which are strongly linked to tumorigenesis. Wang et al. (2019) recently reported that CD8-positive T cells induced ferroptosis in tumor cells. Chen et al. (2017) reported that ATF4, a gene that sensitizes tumor cells to ferroptosis, promotes glioma cell migration. Hong et al. (2017) suggested the involvement that the p53-independent CHOP/PUMA axis in response to ferroptosis inducers, which could play a key role in ferroptotic agent-mediated sensitization to TRAIL-induced apoptosis. Emerging evidence suggests that ferroptosis often shares common pathways with other types of biological functions, including cell death (Bock and Tait, 2019), immune response (Stockwell and Jiang, 2019), and migration (Chen et al., 2017). We found that the risk scores of the signature of the 19 ferroptosis-related genes was negatively correlated to the expression of the well-recognized gene MGMT for TMZ resistance, suggesting the association of ferroptosis with glioma drug resistance. We further found that in glioma cell lines, erastin-induced ferroptosis is closely related to autophagy but not apoptosis because erastin increased the autophagy marker LC3 level but not the apoptosis marker PARP levels. Notably, erastin promoted cell migration in glioma cell lines. Increased cell migration likely occurred prior to the ferroptosis of the glioma cells.

In summary, this study is the first to investigate ferroptotic gene expression patterns in glioma patients and identify their relationship to patient outcome. The ferroptotic signature identified in our study exhibited potential as a biomarker of OS in glioma patients. Understanding the mechanisms underlying ferroptosis and its effect on OS, as well as its implications for the treatment of glioma, can provide insights into the identification of therapeutic targets for glioma.

## Data Availability Statement

All datasets generated for this study are included in the article/Supplementary Material.

## Ethics Statement

This research protocol was approved by the ethical committee of the institutional review board of Capital Medical University and a consent form was signed by each participating patient.

## Author Contributions

HL, CZ, and TJ: conception and design, data analysis and interpretation (statistical analysis), and manuscript writing and revision. HL, HH, and GL: development of methodology. HL, YZ, FW, XL, and KW: data acquisition. All authors read and approved the final manuscript.

## Conflict of Interest

The authors declare that the research was conducted in the absence of any commercial or financial relationships that could be construed as a potential conflict of interest.

## Footnotes

1 http://www.cgga.org.cn

2 https://portal.gdc.cancer.gov

3 https://www.ncbi.nlm.nih.gov/geo/

4 http://gliovis.bioinfo.cnio.es/

5 http://david.abcc.ncifcrf.gov/home.jsp

## Abbreviations

1p/19q: the short arm of chromosome 1 and the long arm of chromosome 19
AKR1C2: Aldo-Keto Reductase Family 1 Member C2
ALOX12B,: Arachidonate 12-Lipoxygenase12R Type
ALOX5: arachidonate 5-lipoxygenase
ALOX5AP: arachidonate 5-lipoxygenase activating protein
ATP5G3: ATP synthase membrane subunit C locus 3
ATRX: alpha thalassemia/mental retardation syndrome X-linked
CBS: cystathionine beta synthase
CCK-8: cell counting kit-8 assay kits
CGCG: Chinese Glioma Cooperative Group
CGGA: Chinese Glioma Genome Atlas
CISD1: CDGSH iron sulfur domain 1
CL: confidence level
DMEM: Dulbecco’s modified Eagle’s medium
DPP4: dipeptidyl peptidase 4
EGFR: epidermal growth factor receptor
EMC2: ER membrane protein complex subunit 2
FANCD2: FA complementation group D2
FBS: bovine serum albumin
G6PD: glucose-6-phosphate dehydrogenase
GBM: glioblastoma multiforme
GCLC: glutamate-cysteine ligase catalytic subunit
GCLM: glutamate-cysteine ligase modifier subunit
GEO: gene expression omnibus
GO: gene ontology
GPX4: glutathione peroxidase 4
GSCs: stemness of glioma stem cells
GSH: glutathione
GSVA: gene set variation analysis
HIF: hypoxia-inducible factor
HMGCR: 3-hydroxy-3-methylglutaryl-CoA reductase
HR: Hazard ratios
HSPB1: heat shock protein beta-1
ICD-O-3,: International Classification of Diseases–Oncology version 3
IDH: isocitrate dehydrogenase
KEGG: Kyoto Encyclopedia of Genes and Genomes
LOXs: lipoxygenases
LPCAT3: lysophosphatidylcholine acyltransferase 3
MGMT: O6-methylguanine-DNA methyltransferase
NCOA4: nuclear receptor coactivator 4
NFE2L2,: nuclear factor erythroid 2 like 2
OS: overall survival
PCD: programmed cell death
PUFAs: polyunsaturated fatty acids
REMBRANDT: The Repository of Molecular Brain Neoplasia Data
ROC: receiver operating characteristic
ROS: reactive oxygen species
SAT1: spermine *N1*-acetyltransferase 1
SIRT2: sirtuin 2
TCGA: The Cancer Genome Atlas
TMZ: temozolomide
TRAIL: TNF-related apoptosis-inducing ligand
U251TR: temozolomide resistant U251MG cells
U87TR: temozolomide resistant U87MG cells
WHO: World Health Organization.
